# Changes of Tissue Reactions on Various HA Filler Under Immune Stress Status: An In Vivo Study

**DOI:** 10.1007/s00266-026-05913-0

**Published:** 2026-06-12

**Authors:** Yin Chen, Youliang Zhang, Weijin Hong, Shengkang Luo

**Affiliations:** 1 The Second School of Clinical Medicine, Southern Medical University, Guangzhou, Guangdong China; 2 Plastic and Reconstructive Surgery Department, Guangdong Second Provincial General Hospital, Guangzhou, 510317 Guangdong China

**Keywords:** HA filler, Immune stress, Tissue reaction, Cross-linking, Anaphylaxis

## Abstract

**Background:**

Post-COVID-19, there has been an increase in reports of anaphylaxis to hyaluronic acid (HA) filler injections.

**Objective:**

This study aimed to investigate the differences in tissue reactions induced by HA fillers produced via various cross-linking technologies under conditions of immune stress.

**Methods:**

A sensitized guinea pig model was utilized to evaluate the anaphylactic responses to different HA fillers. The biological reactions at the injection sites were assessed through hematoxylin and eosin staining. Material properties of the HA fillers were characterized using rheological testing.

**Results:**

Upon injection into sensitized guinea pigs, anaphylactic reactions varied in intensity. The probability of anaphylaxis increased with the degree of cross-linking in the HA fillers, with the reactions predominantly localized to the intradermal injection sites. Histopathological analysis revealed varying degrees of lymphocyte infiltration and other pathological changes across the experimental groups. HA gels produced using different technologies exhibited distinct rheological properties.

**Conclusion:**

The degree of cross-linking and the manufacturing technologies employed in the production of HA fillers significantly impact the occurrence of clinical reactions during immune stimulation. These findings suggest that the administration of HA fillers should be meticulously contemplated in the context of immune stress.

**No Level Assigned:**

This journal requires that authors assign a level of evidence to each submission to which Evidence-Based Medicine rankings are applicable. This excludes Review Articles, Book Reviews, and manuscripts that concern Basic Science, Animal Studies, Cadaver Studies, and Experimental Studies. For a full description of these Evidence-Based Medicine ratings, please refer to the Table of Contents or the online Instructions to Authors www.springer.com/00266.

## Introduction

Following the COVID-19, there has been a marked increase in the incidence of anaphylaxis following fillers injections, particularly those containing hyaluronic acid (HA) fillers [1]. This surge in cases has been linked to both the pandemic and vaccination [2]. Anaphylactic reactions generally occur within a few hours to a few days post-injection and present with symptoms such as erythema, pruritus, and urticaria, which is type I hypersensitivity, mediated by immunoglobulin E (IgE) [3, 4]. The prevailing hypothesis is that the increase in anaphylaxis may be attributed to the activation of the immune system [5].

HA typically does not elicit an immune response [6–9]. To enhance the volume retention of HA products, various manufacturing processes are utilized, including cross-linking technology and the addition of excipients [10–13]. HA produced through different degrees of cross-linking and different processes can result in varying degrees of tissue reactions [14–16]. It has been observed that HA with a higher degree of cross-linking and greater viscoelasticity tends to elicit a more pronounced tissue response [17]. However, it remains unclear whether different cross-linking technologies or the addition of excipients influence allergic reactions under immune stress. A recent review by Pieretti et al. on the use of HA fillers in patients with autoimmune diseases such as scleroderma and SLE highlights a critical dichotomy. Although the pro-inflammatory potential of low molecular weight HA fragments theoretically raises concerns about disease exacerbation, clinical evidence indicates that these treatments can actually improve skin lesions without triggering flares. This discrepancy underscores that the host’s immunologic background is a key determinant of the tissue response to HA. Moreover, the increased incidence of anaphylactic reactions to HA fillers following COVID-19 infection points to a state of generalized immune activation, providing a strong rationale for investigating HA filler safety in other contexts of immune dysregulation, such as allergy [18].

In this study, we conducted a preliminary report investigating the differences in tissue reactions elicited by HA produced using various cross-linking technologies and excipients when implantation into immune-stressed guinea pigs. We focused on acute allergic reactions and compared the observed differences across various injection layers. Initially, we validated using two HA fillers manufactured with identical cross-linking techniques but differing in degree of cross-linking. For comparative analysis, we selected three commercially available HA products with similar clinical profiles: HA-1 manufactured using the TRI-HYAL cross-linking technique [19], HA-2 utilizing the PNET cross-linking technique [20], and HA-3, which incorporated water-soluble cellulose–hydroxypropyl methylcellulose (HPMC) into the BDDE-cross-linked gel formulation to prolong its degradation time [21]. Given that HA-3 contained HPMC, we also compared two HA products with different concentrations of HPMC: HA-3, which contained cross-linked HA and low concentrations of HPMC, and HA-4, which contained non-cross-linked HA and high concentrations of HPMC, to determine whether the inclusion of HPMC introduced additional risks. Concurrently, we characterized the rheological properties of the HA fillers. In animal model selection, the guinea pig sensitization model is widely employed in the study of allergic diseases and the evaluation of drug and medical device sensitization [22–25]. Bovine serum albumin (BSA) can be employed as an antigen to induce an allergic response in animals, activate the immune system, and generate specific IgE antibody, which can serve as one of the evaluation indices [26–28]. We utilized a guinea pig allergy model to simulate the clinical immune activation status. The serum antibody levels and skin conditions of the sensitized guinea pigs were evaluated to determine the optimal timing for HA fillers implantation.

## Materials and Methods

### Materials

Low-cross-linked HA (HA-Low) and high-cross-linked HA (HA-High) gels were prepared and supplied by the Beijing Engineering Lab of Neobiodegradable Materials (Beijing, China). HA-1 was obtained from FillMed (Paris, France). HA-2 was obtained from Dongbang Medical Co., Ltd. (Seoul, South Korea). The medical hydroxypropyl methylcellulose–sodium hyaluronate solution HA-3 (HPMC = 0.75mg/mL) and HA-4 (HPMC = 20mg/mL) were produced by Imeik Technology Development Co., Ltd. (Beijing, China). All gels were provided in prefilled 1 mL glass syringes and sterilized. BSA was purchased from Sevicebio Co., Ltd. (Wuhan, China) and a guinea pig IgE enzyme-linked immunosorbent assay kit was obtained from Zhuocai Biotechnology Co., Ltd. (Shanghai, China).

### Animal Model

The experimental protocol was approved by the Committee (IACUA No. LLSN-204066), and the animals were acclimatized to the experimental conditions for at least one week prior to the initiation of the experiment.

Forty-six 8-week-old male guinea pigs were obtained from Lilaisinuo Biotechnology Co., Ltd. (Sichuan, China) and were housed in a standard environment with temperature of 24±2℃, relative humidity of 40–70% and a 12-h light/dark cycle. The animals had unrestricted access to food and water. Four animals were employed to validate the sensitization protocol, which utilized BSA as the sensitizing agent. On days 1, 3, and 5 following the completion of the acclimatization period, the animals received subcutaneous injections of 0.5mL BSA (10mg/kg). Plasma was collected to assess IgE concentrations prior to the initial sensitization, immediately following the three sensitizations, and on days 7 and 14 post-last sensitization. The animals were euthanized 14 days after the last sensitization, and the lungs and back skin were harvested for histopathological analysis.

### HA Filler Injection

Following the confirmation of the sensitization protocol, sensitized animals were injected with fillers. Seven days post-last sensitization, the animals maintained the sensitization state while avoiding mortality that could result from intense stimulation, and their dorsal hair was removed following isoflurane anesthesia. The animals were allocated into seven groups of six, each receiving subcutaneous and intradermal injections of HA-Low, HA-High, HA-1, HA-2, HA-3, HA-4, or normal saline. The injection volumes were 0.5mL for subcutaneous and 0.1mL for intradermal. Post-injections, animals were monitored biweekly, and anaphylaxis was documented. Common clinical reactions at the injection site included erythema, redness and swelling, and skin ulceration. The incidence rate was calculated as n/N, where n denotes the number of animals displaying allergic reactions and N is the total number of animals within the group.

### Histopathology

All animals were euthanized 14 days post-injection. The implantation and surrounding skin were excised and fixed with 10% neutral formaldehyde. Skin samples were then embedded in paraffin, sectioned into 5-µm-thick sections, and stained with hematoxylin and eosin (H&E). Histopathological changes in the skin at the implant site were examined using a microcamera system and the Pannoramic 250 (3DHISTECH Ltd., Budapest, Hungary). Semi-quantitatively assessments of tissue reactions were conducted using a scale as specified in ISO 10993-6:2016 (Biological evaluation of medical devices—Part 6: Tests for local effects after implantation), which ranging from no reaction to severe reaction:

Score 0.0 to 2.9: minimal or no reaction.

Score 3.0 to 8.9: slight reaction.

Score 9.0 to 15.0: moderate reaction.

Score exceeded 15.1: severe reaction.

### Determination of HA Gel Properties

#### Rheological Measurements

Rheological measurements were performed using DHR-2 rheometer (TA Instruments, New Castle, DE, USA) equipped with parallel plate geometry (plate diameter, 40mm; gap, 1000μm) at 37℃. The G′ (elasticity modulus), G″ (viscous modulus), and tanδ (loss tangent) were determined at a frequency of 1 Hz and an amplitude of 0.1%.

#### Fluidity

**Fig. 1:**
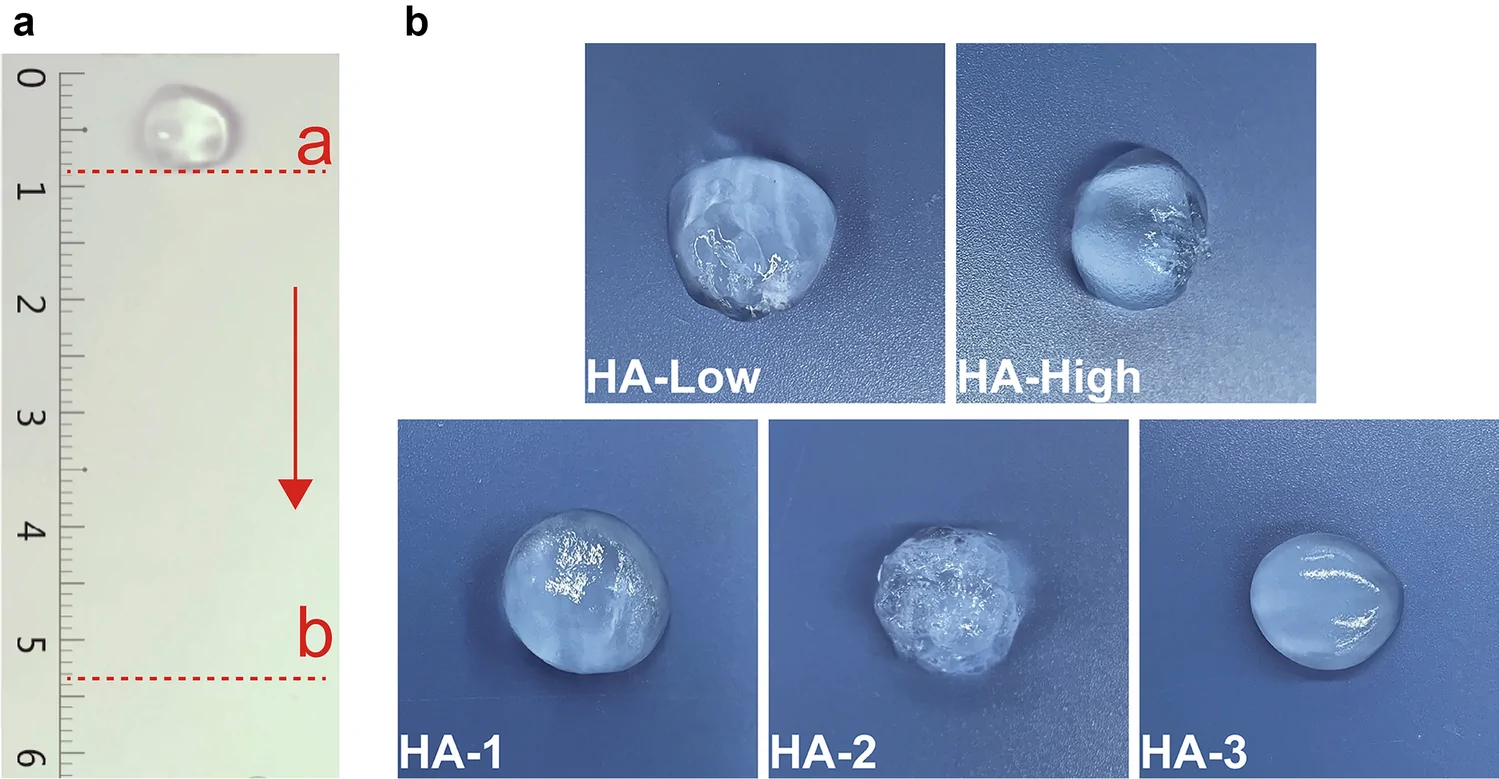
Flow test and photographs of HA gels. (**a**) Schematic diagram of the flow test. The dotted line was the starting position. The gel sample was allowed to flow naturally (in the direction indicated by the arrow) for 30 min, and the final position was determined as dotted line b. Flow distance (mm) = b-a. (**b**) Photos of the five gels. HA-Low, low cross-linking degree HA gel. HA-High, high-cross-linking degree HA gel. HA-1, HA-2, and HA-3 represent HA products produced by the three cross-linking technologies of TRI-HYAL, PNET, and HA and HPMC synergistic technology

A sample of gel (0.5mL) was injected layer-by-layer onto a circular polytetrafluoroethylene plate with a diameter of 10mm. The plate was placed vertically at 90° (Fig. 1a), and the flow distance within 30min was recorded for each sample.

#### Extrusion Force

The extrusion force of the prefilled syringes was measured using UTM6202 tensile tester (Sansi, Shanghai, China) at a constant speed of 30 mm/min and 27G needle (Wego Medical, Weihai, China).

#### Swelling Factor (Gel Fluid Uptake)

A 500-mesh sieve (8×8cm square folded into a square slot 4×4×2cm) was labeled m_0_. Accurately weighed 0.2~0.5g gel (labeled as m_1_) was placed on the sieve, which was then placed on a plate with 30mL normal saline. The sample was allowed to infiltrate completely for 30min. The sieve was removed with the sample, and filter paper was used to absorb any remaining liquid, which was weighed as m_2_. The swelling factor was calculated according to the formula (m_2_-m_0_)/m_1_.

#### Cohesion

Cohesion was measured using UTM6202 tensile tester at a constant speed of 7.5mm/min and 25G needle (Wego Medical). Once a constant force was achieved, 10 drops were collected, and the average weight of each drop was calculated.

Rheological measurements were not possible for HA-4 due to its non-gelatinous nature.

#### Statistical Analysis

Data were analyzed and visualized using GraphPad Prism software version 8 (GraphPad Software, San Diego, USA). Between-group differences in categorical variables were assessed using Fisher’s exact tests. The normality of the histological evaluation score within each group was assessed, and differences in scores across injection layers were evaluated using t-tests. Intergroup differences were analyzed using one-way analysis of variance (ANOVA). Each experiment was repeated at least three times. All data are presented as mean ± standard (SD).

## Results

### Animal Model

**Fig. 2:**
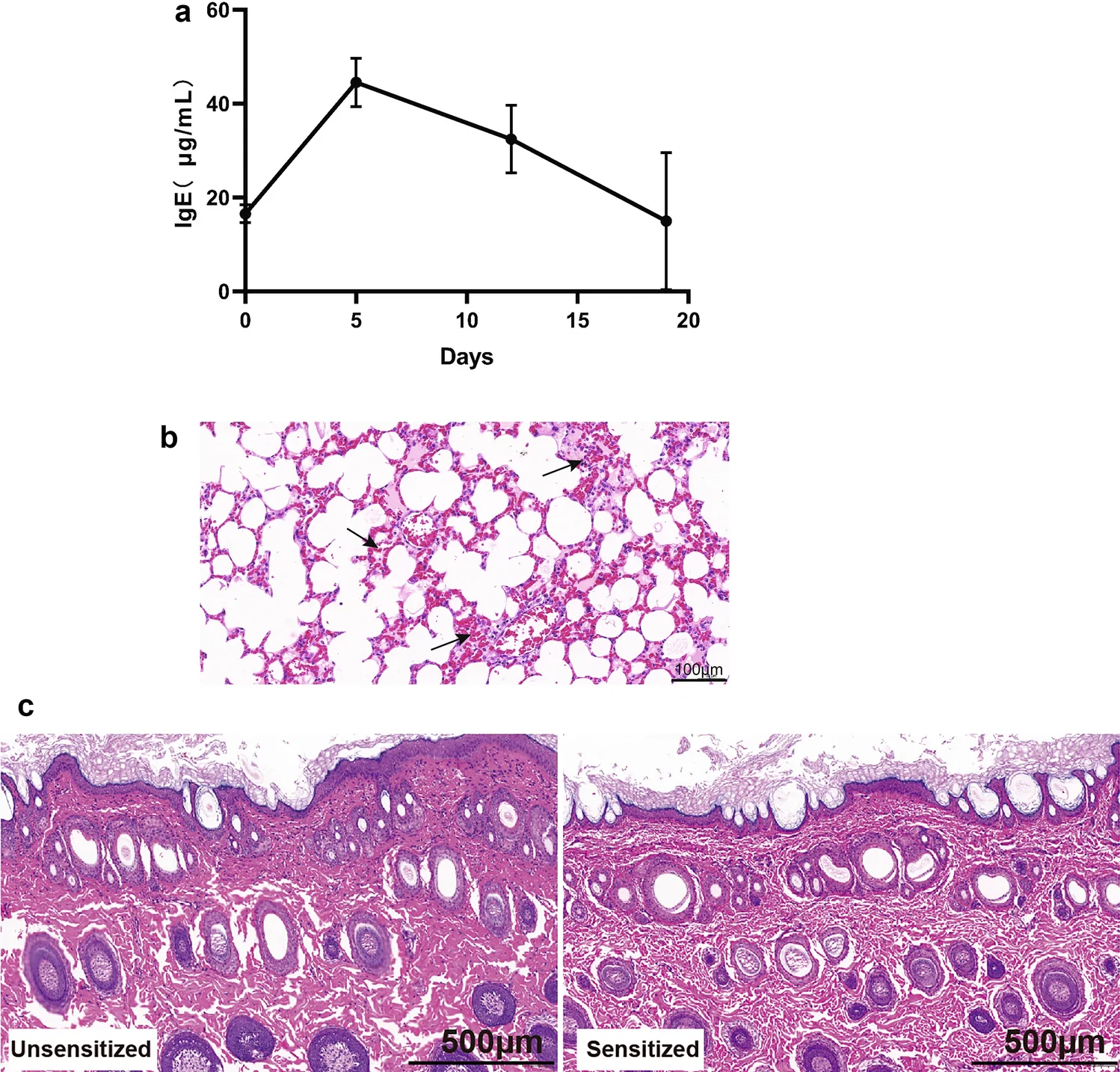
Animal model and implantation time selection. (**a**) The temporal dynamics of serum IgE concentrations were quantitatively assessed both pre- and post-BSA sensitization. The serum IgE concentration can be maintained at a high level for 2 weeks after sensitization. (**b**) Alveolar thickening and congestion in the lungs of sensitized guinea pigs (black arrow). Lung tissues were collected 14 days following the completion of three sensitization sessions for histological examination using H&E staining to assess the allergic status of the guinea pigs. (**c**) H&E staining of the skin tissues of unsensitized and sensitized guinea pigs. Comparative analysis of dorsal skin characteristics between sensitized and non-sensitized guinea pigs was conducted to verify that the sensitization procedure itself did not induce any significant alterations in the dorsal skin morphology

The serum IgE levels of the guinea pigs were elevated following sensitization with BSA, reaching peak levels after three rounds sensitizations. These elevated IgE levels persisted for two weeks after the last sensitization (Fig. 2a). After three rounds of sensitization, animals exhibited symptoms including nose scratching and wheezing. Histopathological analysis revealed thickening of the pulmonary alveolar septum, capillary hemorrhaging, and lymphocyte infiltration (Fig. 2b). During the observation period, no dermal abnormalities were observed in the guinea pigs. H&E staining of the back skin tissue, which is planned as injection site, did not reveal any significant pathological differences between sensitized and unsensitized animals (Fig. 2c).

### General Observation

**Fig. 3:**
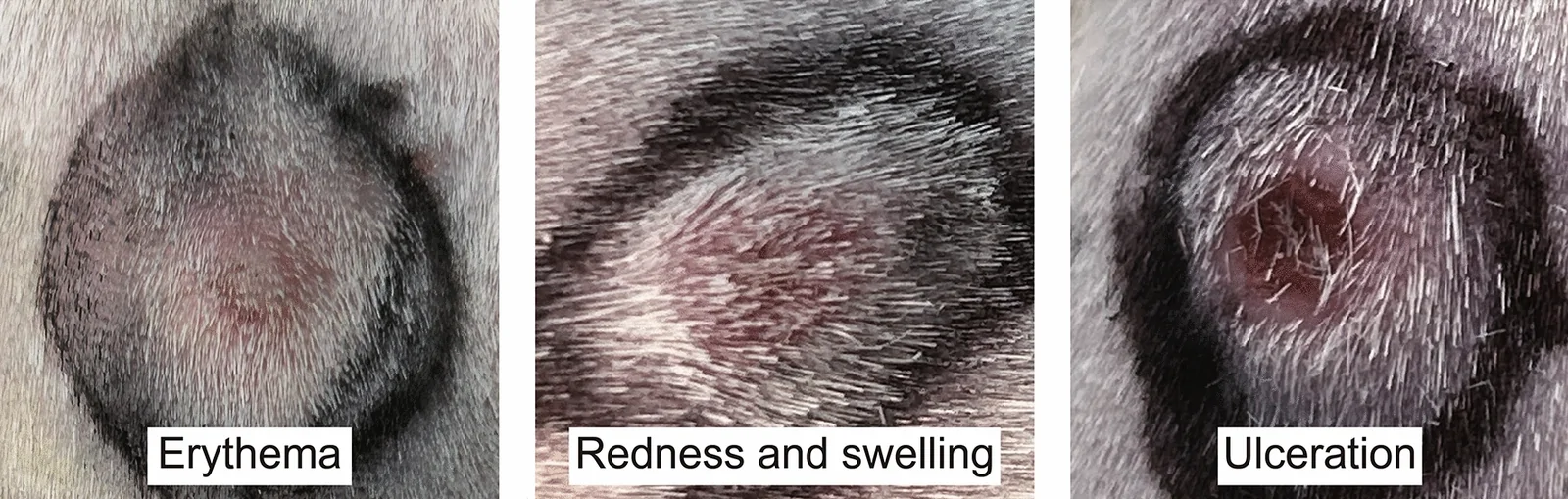
Clinical reactions after injection of different HA fillers. Following the implantation of HA fillers, local skin reactions at the injection site were monitored and documented. The most frequently observed clinical reactions included erythema, redness and swelling, and ulceration

Anaphylaxis probabilities for each group are presented in Table 1. No anaphylaxis was observed at the implantation site in the saline group. Clinical reactions were observed across all experimental groups, to different ratio. The incidence of anaphylaxis was 16.7% (1/6) in the HA-Low group, 33.3% (2/6) in the HA-High group, and 100% (6/6), 50% (3/6), 33.3% (2/6), and 33.3% (2/6) in the HA-1, HA-2, HA-3, and HA-4 groups, respectively. The most common observed anaphylaxis was erythema and ulceration (Fig. 3). Although no statistically significant differences were found, a higher frequency of clinical reactions was observed at the intradermal compared to the subcutaneous layers.

**Table 1:** Anaphylaxis after hyaluronic acid injection in each experimental group

Events(n/N) groups	Saline		HA-Low		HA-High		HA-1*		HA-2		HA-3		HA-4	
Layers	Int	Sub	Int	Sub	Int	Sub	Int	Sub	Int	Sub	Int	Sub	Int	Sub
Erythema	0/6	0/6	1/6	0/6	0/0	0/6	5/6	0/6	2/6	0/6	1/6	0/6	1/6	1/6
Redness and swelling	0/6	0/6	0/6	0/6	1/6	0/6	0/0	0/6	0/0	0/6	0/0	0/6	0/0	0/0
Ulceration	0/6	0/6	0/6	0/6	2/6	0/6	6/6	0/6	2/6	0/6	2/6	0/6	2/6	0/0
Total	0/6	0/6	1/6	0/6	2/6	0/6	6/6	0/6	3/6	0/6	2/6	0/6	2/6	1/6
	0/6		1/6		2/6		6/6		3/6		2/6		2/6	

During the 14-day period following hyaluronic acid (HA) implantation, all clinical reactions observed at implantation sites were meticulously documented. N, number of animals per group; n, number of events. Data includes intradermal and subcutaneous injection site anaphylaxis. If two or more anaphylaxis occurred in one animal, they were combined as one animal. Int, intradermal; Sub, subcutaneous. * denotes significant differences in the incidence of clinical reactions among various injection layers within the same sample group. * indicates *p* < 0.05.

### Histopathology

**Fig. 4:**
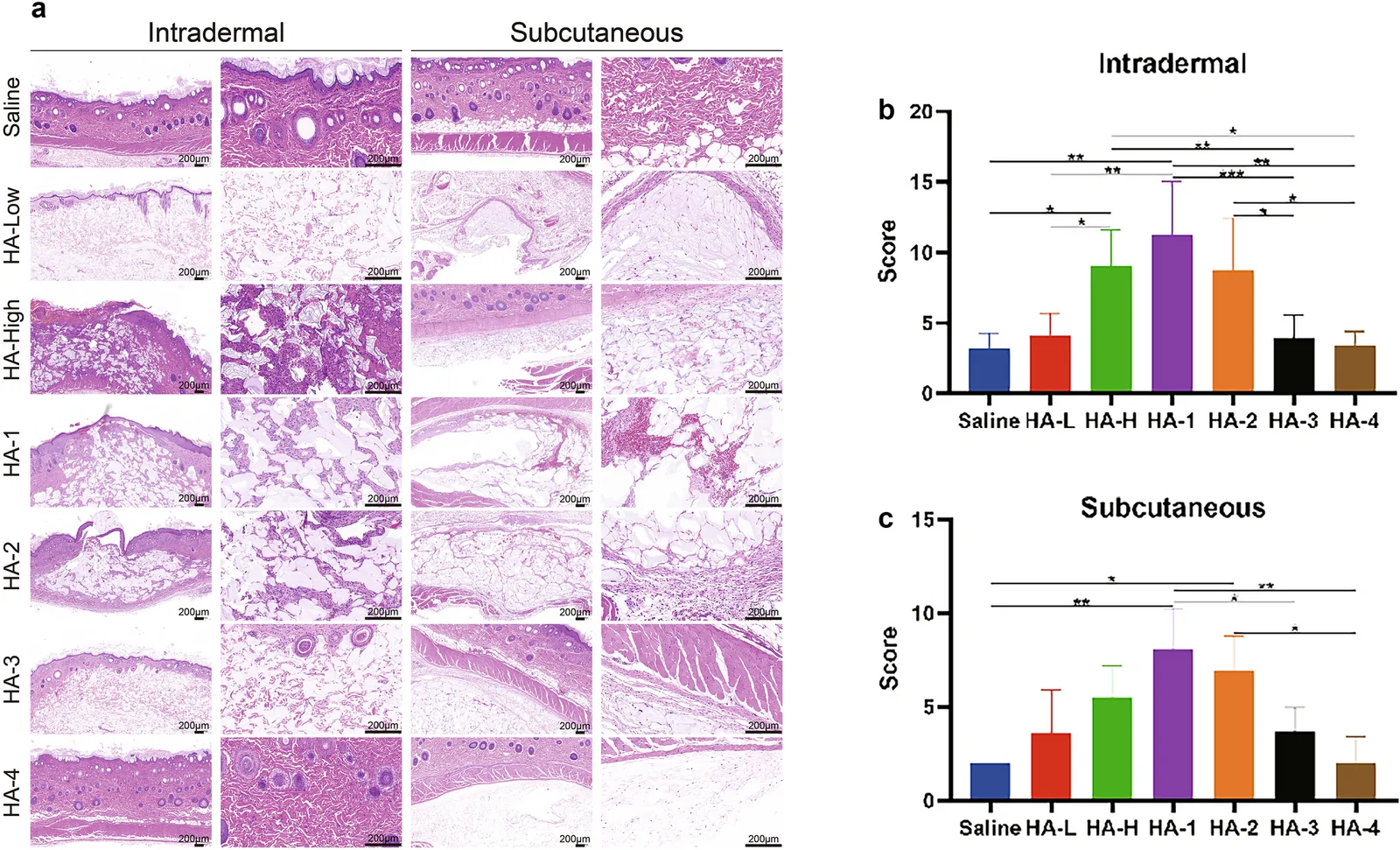
After 14 days post-implantation, skin tissue specimens were harvested from the injection sites and subjected to histopathological analysis through H&E staining to evaluate intergroup differences in tissue reactions. (**a**) Histopathological staining of implantation sites from guinea pigs receiving different hyaluronic acid fillers. Histological evaluation score of (**b**) intradermal layer and (**c**) subcutaneous layer, conducted in accordance with ISO 10993-6, Scores for each group are those without baseline deduction; * denotes *p* < 0.05, ** denotes *p* < 0.01, and *** denotes *p* < 0.001

H&E staining results are depicted in Fig. 4a. No pathological changes were observed in the saline group. In HA-Low group, minimal inflammatory cell infiltration was noted at both intradermal and subcutaneous injection sites. In HA-High group, distinct implant morphology was observed at the intradermal site, accompanied by a high number of inflammatory cells, phagocytes, histiocytic infiltrations, and extensive red blood cell exudation. Inflammatory cell infiltration was also observed at the subcutaneous injection site. Red blood cell exudation was also observed at the intradermal injection site in HA-1 and HA-2 groups, along with a large number of lymphocytes, phagocytes, and granulocytes around the implant. Lymphocyte and eosinophil infiltration of the subcutaneous injection site was accompanied by red blood cell exudation. Only slight lymphocyte infiltration was observed at the intradermal and subcutaneous injection sites in HA-3 group. Implants in HA-4 group were not visible, and no significant inflammatory cell infiltration or pathological changes were noted. The scores of tissue reactions following intradermal and subcutaneous injections of HA-Low, HA-3, and HA-4 ranged from 0.0 to 1.7, indicating no or minimal reaction. In contrast, HA-High, HA-1, and HA-2 exhibited slight tissue reactions (3.5 to 8.0). Similarly, tissue responses were more pronounced at the intradermal injection layer compared to the subcutaneous injection layer, as evidenced by greater inflammatory cell infiltration and higher tissue response scores. Significant differences were only observed in HA-High group (p < 0.01) and HA-1 group (p < 0.05). The reaction scores of each group are shown in Table 2, Fig. 4b and c.

**Table 2:** Skin inflammatory cell infiltration scores at the injection site of each experimental group

Score groups	Saline	HA-Low	HA-High**	HA-1*	HA-2	HA-3	HA-4
Intradermal	0.0	0.9	5.9	8.0	5.5	0.7	0.2
Subcutaneous	0.0	1.6	3.5	6.1	4.9	1.7	0.0

After 14 days post-implantation, skin tissue specimens were harvested from the injection sites and subjected to histopathological analysis through H&E staining to evaluate intergroup differences in tissue reactions. Histological evaluation score of intradermal layer and subcutaneous layer, conducted in accordance with ISO 10993-6. The scores of each group after baseline were subtracted; the saline group score was used as the baseline. The scores before deducting the baseline can be viewed in Fig. 4 B and C. * denotes significant differences in scores among various injection layers within the same sample group. * indicates *p* < 0.05, and ** indicates *p* < 0.01.

### Determination of HA Gel Properties

All five gels were colorless and transparent, as depicted in Fig. 1b. The physical and chemical properties of the gels are detailed in Table 3. Among the gels evaluated, the gel with the highest G′ was HA-High (1462Pa). HA-Low (66Pa) and HA-1 (93Pa) had lower G′. Similarly, the HA-High gel exhibited a lower flow distance (3mm), whereas HA-1 displayed a higher flow distance (135mm).

**Table 3:** Rheological properties of the hyaluronic acid gels

Product name	HA (mg/mL)	G′ (Pa)	G″ (Pa)	Tan δ	FD (mm)	F_max_ (N)	F_min_ (N)	F_ave_ (N)	SwF	DW (mg)
HA-Low	20	66	14	0.214	18.5	23.52	22.53	23.10	2.4	23.82
HA-High	20	1462	368	0.252	3.0	24.85	22.63	23.49	1.6	10.14
HA-1	20	93	34	0.366	135.0	10.57	8.06	8.43	3.0	9.56
HA-2	24	314	42	0.133	5.2	22.06	20.75	21.45	3.1	12.55
HA-3	19	213	47	0.222	14.5	16.91	16.53	16.67	1.3	16.09

The rheological characteristics of five distinct HA gel formulations were evaluated. *G′* elastic modulus, *G″* viscous modulus, *tan δ* the loss tangent, *FD* flow distance, *F*_*max*_ maximum extrusion force, *F*_*min*_ minimum extrusion force, *F*_*ave*_ average extrusion force, *SwF* swelling factor, *DW* drop weight

The HA-Low (22.53N to 23.52N), HA-2 (20.75N to 22.06N), and HA-High (22.63N to 24.85N) required greater extrusion forces, whereas HA-3 (F_max_=16.91N) and HA-1 (F_max_=10.57N) required significantly lower forces.

Swelling factor results indicated that the HA-3 (1.3) and HA-High (1.6) had lower capacities to absorb additional fluid. In contrast, the HA-Low (2.4), HA-2 (3.1), and HA-1 (3.0) demonstrated greater fluid uptake.

Cohesion data revealed that the HA-Low (23.82mg) and HA-3 (16.09mg) exhibited higher cohesion, whereas the HA-2 (12.55mg), HA-1 (9.56mg), and HA-High (10.14mg) had lower cohesion values.

## Discussion

Early-onset acute inflammatory or allergic reactions associated with HA fillers is generally rare, typically presenting as IgE-mediated type I hypersensitivity reactions [29]. However, the COVID-19 and subsequent vaccination campaigns have led to widespread immune system activation across populations, resulting in an increased incidence of HA filler-related adverse events, including both immediate and delayed inflammatory reactions [30]. To investigate the tissue response to HA fillers under immune stress condition, we utilized guinea pigs, chosen for their sensitivity and the similarity of their skin with humans, and their frequent use in sensitization evaluation [31]. Serum IgE levels, allergic symptoms observed in the guinea pigs, and histopathological examination confirmed successful sensitization.

In this study, we examined the tissue response of HA-Low and HA-High, as well as commercially available facial fillers HA-1, HA-2, HA-3, and HA-4, in immune-stressed guinea pigs. We compared their rheological properties. Animal experimental results indicated that injection of all of the HA fillers into sensitized guinea pigs elicited varying degrees of clinical reactions resembling clinical symptoms. Notably, these reactions were more frequent following the implantation of HA-1 and HA-2 in our model. H&E staining results were consistent with clinical reactions, with HA-Low and HA-3 inducing a weaker tissue response, while HA-High, HA-1, and HA-2 caused greater responses. No significant inflammatory cell infiltration was observed following HA-4 injection. In all HA groups, intradermal injection induced a more reactive tissue response, higher reaction scores, and more frequent clinical reactions, although significant differences were observed in only a few groups, potentially due to the limited sample size. The comparative analysis of rheological properties among different HA fillers revealed significant variations, despite their intended use for facial volume improvement.

Our initial comparison of two HA produced using the same technology but with different cross-linking degrees revealed a positive correlation between the degree of cross-linking and the probability of anaphylaxis and the severity of tissue reactions. Consistent with this, a previous study reported that a high degree of HA cross-linking leads to more clinical adverse reactions [14, 32]. The increased degree of cross-linking necessitates a higher amount of BDDE, though is generally considered safe [33–35]. However, under conditions of immune stress, this may result in additional clinical responses. Moreover, a higher degree of cross-linking also results in higher G′ value, indicating “stiffer” physical properties [17, 36, 37], will further increases the likelihood of anaphylaxis.

To assess the impact of different cross-linking technologies or the addition of excipients on allergic reactions, we simultaneously compared three HA products for facial volume improvement, all of which possess strong volume augmentation and long maintenance effects [38]. Following the implantation of HA filler in sensitized guinea pigs, HA-1 was associated with a higher incidence of allergic reactions, as evidenced by a large number of infiltrating inflammatory cells. HA-1 utilizes TRI-HYAL technology to chemically cross-link HA with different molecular weights and mix it with non-cross-linked HA [18]; achieving the volume augmentation while also having low viscoelasticity and extrusion pressure. HA-2 uses PNET technology to cross-link interspersed HA molecular chains with BDDE, resulting in a higher elastic modulus and a granular gel feel, which provides superior contouring. In animals implanted with HA-2, more clinical and tissue reactions were observed. Among the three evaluated products, HA-3 demonstrated a milder tissue reaction. HA-3 employs HA and HPMC synergistic technology to enhance the rheological properties of HA gels, incorporating HPMC molecular chains into the HA gel matrix to reduce the need for cross-linking agents and increase the retention time. The inclusion of HPMC allows HA-3 possess sufficient physical properties to meet clinical application requirements even with low-cross-linked HA. Our experimental findings showed that HA-3 demonstrated lower levels of inflammation and fewer allergic reactions in the guinea pig sensitization model.

HPMC has been less commonly used in fillers, and few studies have addressed its safety. To determine whether HPMC introduces additional risk factors, we conducted a comparative analysis between HA-3 (0.75mg/mL) and another HA product (HA-4, 20mg/mL). The findings indicated that the incidence of allergic reactions for both HA-3 and HA-4 was 2/6. H&E staining of HA-3 showed a minor presence of inflammatory cells at the injection site, whereas HA-4 exhibited no inflammatory cell infiltration at day 14. Both general observations and histopathology evidence revealed that elevated concentrations of HPMC were not associated with increased incidence of anaphylaxis or enhanced tissue reactions.

Additionally, the comparison of clinical reactions at different injection layers revealed that a greater number of clinical reactions were noted at the intradermal layers. This highlights the importance of matching HA products to appropriate injection layers. Previous studies have similarly reported this phenomenon [39], attributing it to the dense dermal tissue requiring softer and more diffusible HA product [40]. Notably, the differences in tissue reactions induced by varying cross-linking technologies were amplified in the sensitized guinea pig model. While subcutaneous injections of different HA products did not exhibit statistically significant differences in clinical observation outcomes, histopathological analysis revealed notable more subtle distinctions. The incidence of anaphylaxis following HA fillers correlates with the intensity of the tissue reaction, emphasizing the need for careful consideration of these factors in clinical practice. As also noted by Pieretti *et al*., the use of HA fillers in patients with autoimmune inflammatory diseases remains clinically controversial [18]. In light of our findings, the cross-linking technology of fillers may be a relevant consideration when selecting products for individuals with altered immune status. However, further studies in disease-specific models and clinical cohorts are warranted to validate these findings.

Utilizing a guinea pig sensitization model, we analyzed variations in tissue reactions at different layers following the injection of HA with distinct cross-linking technologies or addition excipients, revealing correlations between cross-linking technology or excipients and the incidence of anaphylaxis.

Our study has certain limitations. Firstly, the animal model simulates only a subset of immune stress states and does not fully reflect the entire clinical scenario [41–44]. Secondly, our comparison was limited to four commercially available products with distinct cross-linking technologies. Further research is needed to investigate other representative cross-linking technologies used in HA fillers, such as VYCROSS and NASHA [45–48]. Additionally, the effects of single versus combined or multiple injections also require consideration. The study sample size necessitates augmentation to yield more rigorous scientific outcomes or conclusions. Therefore, further research is warranted. Furthermore, whether similar results would be observed with other currently popular filler materials, such as poly-L-lactic acid (PLLA) or calcium hydroxylapatite (CaHA), warrants further investigation. Nonetheless, the guinea pig sensitization model was chosen to investigate tissue reactions to HA fillers from various cross-linking technologies, providing a reference for future research.

## Conclusion

This study indicate that the cross-linking technology or addition of excipients in HA products can influence the occurrence of anaphylaxis under conditions of immune stress. Specifically, HA products with lower degrees of chemical cross-linking exhibited reduced reaction incidence. Additionally, a more superficial injection layer may exacerbate tissue reactions. In clinical applications, the administration of HA fillers must be carefully considered in the context of immune stress, balance aesthetic outcomes with patient safety.

## Data Availability

The data underlying this article will be shared by the corresponding author upon reasonable request.
